# “One-time interventions, it doesn’t lead to much” – healthcare provider views to improving sexual and reproductive health services for young migrants in Sweden

**DOI:** 10.1186/s12913-022-07945-z

**Published:** 2022-05-18

**Authors:** Veronika Tirado, Siri Engberg, Ingrid Siösteen Holmblad, Susanne Strömdahl, Anna Mia Ekström, Anna Karin Hurtig

**Affiliations:** 1 Department of Global Public Health, Karolinska Institutet, Stockholm, Sweden; 2 Department of Medical Sciences, Infectious Medicine, Uppsala University, Uppsala, Sweden; 3 Department of Infectious Diseases, Venhälsan, Södersjukhuset, Stockholm, Sweden; 4 Department of Epidemiology and Global Health, Umeå University, Umeå, Sweden

**Keywords:** Health workforce, Healthcare providers, Sexual and reproductive health and rights, HIV, Sweden, Migrants, Youth, Access to healthcare

## Abstract

**Background:**

Sexual and reproductive health and rights (SRHR) is an important aspect for young people. In Sweden, young migrants often encounter barriers to accessing and using sexual and reproductive health (SRH) services, despite that these services are free of charge for young people (ages 15–25). Healthcare providers’ views and best practices are of great importance for improving the utilisation of existing SRH services, particularly for young people. This study aims to understand healthcare providers’ experiences and perspectives on barriers to SRHR among young migrants and their suggestions for strategies to improve the provision of SRH services to this group.

**Methods:**

Midwives, counsellors and nurses with at least five years of professional experience within SRHR were reached through a purposeful sample at primary care clinics, specialised clinics and youth-friendly clinics, which provide SRH services to migrant youths in Stockholm. Twelve interviews were conducted from May 2018 to February 2020. Qualitative content analysis was performed.

**Results:**

The analysis identified one theme: Improving the fragmentation in the SRH services, and four sub-themes: 1. Being unaware of SRHR; 2. Creating trust and responsive interactions; 3. Communicating in the same language; and 4. Collaborating to build bridges. The barriers included distrust in the healthcare system, socio-cultural norms surrounding SRHR, incomplete translations, and a need for long-lasting collaboration with SRH services and other range of services for migrants. The strategies for improvement as suggested by participants included involving existing cultural groups and organisations to enable trust, consistent and dependable interpreters, a streamline of SRH services with other healthcare staff and health facilities, and collaborations with homes designated for young migrants and language schools for a direct linkage to service providers.

**Conclusions:**

Findings indicate that there are fragmentations in SRH services, and these include lack of knowledge about SRHR among migrant youth, language and communication barriers, and a lack of structure needed to build dependable services that go beyond one-time interventions. While initiatives and strategies from healthcare providers for improvement of SRH services exist, the implementation of some strategies may also require involving the regional and national decision-makers and multi-stakeholders like communities, civil society and young migrants themselves.

## Background

The key challenges that unfold with sexual and reproductive health (SRH) services for youth, in particular for migrant youth, are to ensure access, provide quality care and health-related information that is relevant and adapted to those most in need [1, 2]. Among the targets for the United Nations’ Sustainable Development Goals (SDG) [3, 4], ensuring access to SRH services, such as contraceptives, safe abortion, prevention and counselling for sexually transmitted infections (STI) is one of the goals related to sexual and reproductive health and rights (SRHR) [5]. Migrant youth in their destination countries and communities often experience policy and legal restrictions, such as language requirements which further limits their ability to articulate their SRHR, practice preventive-health behaviours and utilise essential health services [6, 7].

In 2015, more than 1 million migrants sought refuge in Europe. In Sweden, there were nearly 160,000 asylum seekers, predominately from Syria, Afghanistan, and Iraq [8, 9]. Of those asylum seekers in Sweden, there were 35,000 unaccompanied young refugees, mainly boys (94%) aged 13 to 17 years [10]. European countries have national health policies in place to ensure the safety and access to healthcare services for asylum seekers and migrants [11]. One key priority is to guarantee the human right to health by acknowledging their specific health needs and addressing these issues through actions, such as providing health coverage and social protection [12, 13]. Asylum seekers in Sweden are entitled to emergency healthcare, dental care, and healthcare that cannot be postponed, as well as to an interpreter when visiting the healthcare staff [14]. Migrant youth under the age of 18 have the same right to subsidised healthcare services as those who are permanent residents. In addition, under-aged asylum seekers have the right to attend school in Sweden. Language learning and age-related sexual education are, to a great extent, integrated in the schools [15]. SRH services such as contraceptive counselling and testing for STIs are free of charge in Sweden and tend to be provided by midwives in prenatal clinics or youth-friendly clinics [16]. Information about the youth-friendly clinics and basic sexual health information is available to migrants in Sweden in different languages by web-based national websites [17, 18]. Temporary homes are also offered to asylum seekers in Sweden, and these are often allocated by the municipality. Among the different types of housing, the homes for care or residence has separate homes for children and for people who are over 18 years old [19]. Asylum seekers are also invited to take part in two-year introduction programmes focused on language learning through basic Swedish courses that are free of charge for all migrants, and a civic orientation course and labour activities that are organised by the Swedish Public Employment Service [20, 21]. However, explicit information about SRHR in Sweden is not part of these introduction programmes, and young migrants are prompted to get this kind of information through other sources such as their peers at school, via the internet or when visiting SRH services like youth-friendly centres [17, 22, 23].

Migrants, especially the youth, have varied and complex health needs. A major challenge is that they often have difficulties navigating the healthcare system due to language barriers and lack of parental support, which may lead to under-utilisation of SRH services [24, 25]. Social and cultural norms like shared beliefs often exclude men who have sex with men and lesbian, gay, bisexual, trans, queer or questioning people (LGBTQ) from the prevailing social groups, and sometimes make open discussion about sex and sexuality difficult among young migrants [26]. Consequently, reproductive health issues including STIs, unplanned pregnancies and other health concerns may not be addressed with family members and healthcare providers [7]. Migrant youth in Sweden have reported unfamiliarity to the healthcare system, long waiting times to get an appointment for SRH services, and difficulties with the language and costs for transportation to access these services [27]. Research on migrant youths has focused mostly on evidence of their unmet needs and own views of services [28–30] and less on health providers perspectives [31, 32]. A study in the UK found that healthcare providers encountered challenges to creating a fit between recently arrived migrants and the clinical setting. For example, it was problematic from the healthcare provider’s view to match and align trust due to different expectations by the health personnel and migrant patients [33]. Midwives in Sweden have reported difficulties to counsel migrants regarding contraception and the need to adapt the contraceptive counselling for each interaction and situation [34]. Other Scandinavian studies showed that healthcare professionals have experienced language barriers with migrants and have a preference for professionally-trained interpreters rather than family members as interpreters [35–37].

Healthcare providers act as gatekeepers to essential health services such as antenatal care, postnatal care and delivery, and fertility assistance programmes [38]. The healthcare provider views are of great importance in a rapidly changing healthcare environment. There is little research describing the healthcare providers’ views of migrant youths’ unmet needs and health service utilisation in relation to SRH services, proposed alternatives and solutions [5]. We aimed to understand healthcare providers’ experiences and perspectives on barriers and their strategies for improvement of providing SRH services to young migrants.

### Conceptual framework

We initially applied the Andersen’s expanded health behaviour model to understand how and why people use healthcare services [39]. There are three main clusters in this framework: the predisposing factors like age and individual characteristics; the enabling factors, such as financial resources and conditions to serve as healthcare utilisation; and the need factor, which differentiates between the persons’ perceived need for health services, such as general health-state, and professionally evaluated need for care, such as measurements of the health status like STI testing [39]. We then used the Socio-ecological framework to highlight barriers and facilitators for the SRHR among young migrants. The framework aided our research objective since it focuses on multi-cultural backgrounds across the socio-ecological spectrum, from the individual to system levels [7, 40]. For example, migrant youth may have past experiences and misconceptions, such as poor risk perceptions (e.g. risk-taking behaviours) at an individual level that could present challenges to healthcare providers [7].

## Methods

### Study setting and design

The Swedish healthcare system is mainly publicly tax-funded, although private healthcare also exists [16]. The primary care clinics in Sweden are often allocated in practices, and there are about 1200 primary healthcare centres [41]. Specialised care clinics, including antenatal care, are organised in both out-patient clinics and hospitals. There are 262 youth-friendly clinics in Sweden, of which 17 are located in Stockholm [18]. Nearly 55,000 foreign-born youths (15–24 years) reside in Stockholm [42]. We conducted 12 semi-structured interviews with healthcare providers to gain a rich understanding of their perceptions of providing SRH services to Stockholm-based migrant youth between the ages of 15 and 25 years. The interviews were conducted from May 2018 to February 2020, and the participants were employed in the Swedish healthcare system.

### Study participants and recruitment

Healthcare facilities were listed based on location and type of health service provided, such as primary care clinics, specialised care clinics including antenatal care, testing centres for STI, and youth-friendly clinics. The research team revised the list, and a letter containing information about the study was sent to 21 healthcare facilities to invite participants for an interview. We purposefully selected the health facilities that focused on providing SRH services and to get representation from areas with various socio-economic contexts. We reached 11 health facilities and conducted 12 interviews with healthcare providers, 10 of whom were interviewed face-to-face and two via telephone (Table 1). The participants had a profession as either nurse, midwife or counsellor. Two of the participants were male. The age range was between 39 and 61 years old, and the participants’ years of professional experience ranged from 5 to 34 years.

**Table 1:** Description of the participants

Code	Profession	Gender	Health facility	Length of working experience	Work experience abroad
1	Nurse	Female	Primary care clinic	30 years	No
2	Nurse	Female	Primary care clinic	13 years	No
3	Midwife	Female	Specialised clinic	20 years	No
4	Midwife	Female	Youth-friendly clinic	34 years	No
5	Midwife	Female	Youth-friendly clinic	17 years	No
6	Midwife	Female	Youth-friendly clinic	5 years	No
7	Counsellor	Female	Youth-friendly clinic	13 years	No
8	Nurse	Female	Specialised clinic	18 years	No
9	Nurse	Male	Specialised clinic	20 years	Yes
10	Midwife	Female	Youth-friendly clinic	10 years	Yes
11	Counsellor	Female	Specialised clinic	8 years	No
12	Counsellor	Male	Youth-friendly clinic	21 years	No

### Data collection

The interview guide was developed with a focus on current barriers and strategies or possible solutions in the healthcare system that prevent effective uptake of different SRH services. The SRHR domains for the interview guide included HIV and STI testing, contraception, and early pregnancy screening or abortion services. The interviews were held in the participants’ preferred language, either Swedish or English. The duration of the interviews was between 30 min and 2 h. We conducted the interviews until we reached data saturation [43]. We asked the participants to discuss from their own experience and focus on young people who had recently arrived (in the last two years) without considering the reason for migrating or their legal status in Sweden. This allowed the participants to describe their experiences of working with undocumented migrants, young migrants with a temporary residency permit or seeking asylum, and young migrants who were permanently residing with their families in Sweden.

### Data analysis

**Fig. 1:**
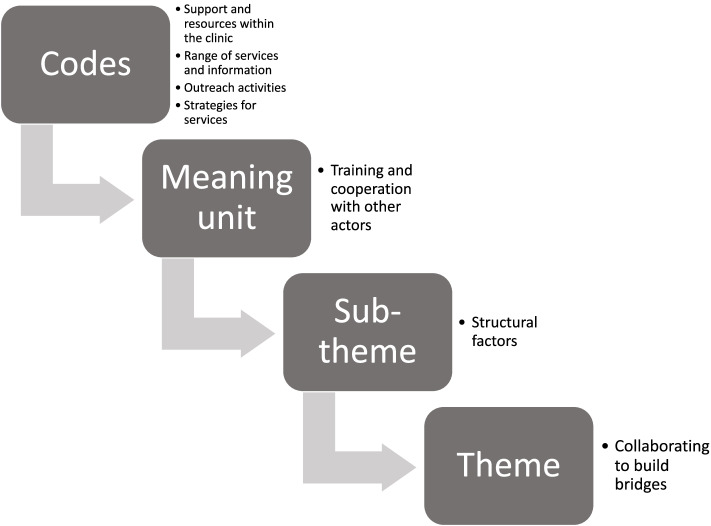
A description and example of the process of analysis

We conducted a qualitative content analysis [44] using the Framework Method [45] and supported by the software MAXQDA 2018 (180830) Version 18.2.4. The interviews were transcribed in its original language to conserve accuracy and meaning. Familiarisation with data took place, and an inductive approach was applied to generate codes and meaning units as expressed by the participants. Researchers VT, SE and ISH independently coded all interviews and developed the analytical framework, on which SS, AME and AKH provided feedback. Differences were resolved through discussions among all researchers. VT charted the data into a framework matrix and the data was additionally interpreted by writing memos for each of the four concepts of categories as possible sub-themes. VT discussed the memos with all researchers involved and verified the process of analyses (Fig. 1). There were four sub-themes and one overarching theme that outlined the barriers perceived by healthcare providers, and we highlighted their experiences and strategies for improvement to SRH services.

### Ethical considerations

We provided information about the study to all participants. We obtained both verbal and written informed consent, and the participants were informed that participation was voluntary with the possibility to opt-out at any time without explanation. The interviews were coded and made anonymous using a number and reference code. All materials from the interviews were securely stored, and access was limited to only the research team. Approval for this study was received from the Swedish Ethical Review Authority (Dnr: 2017/2030–31).

## Results

**Fig. 2:**
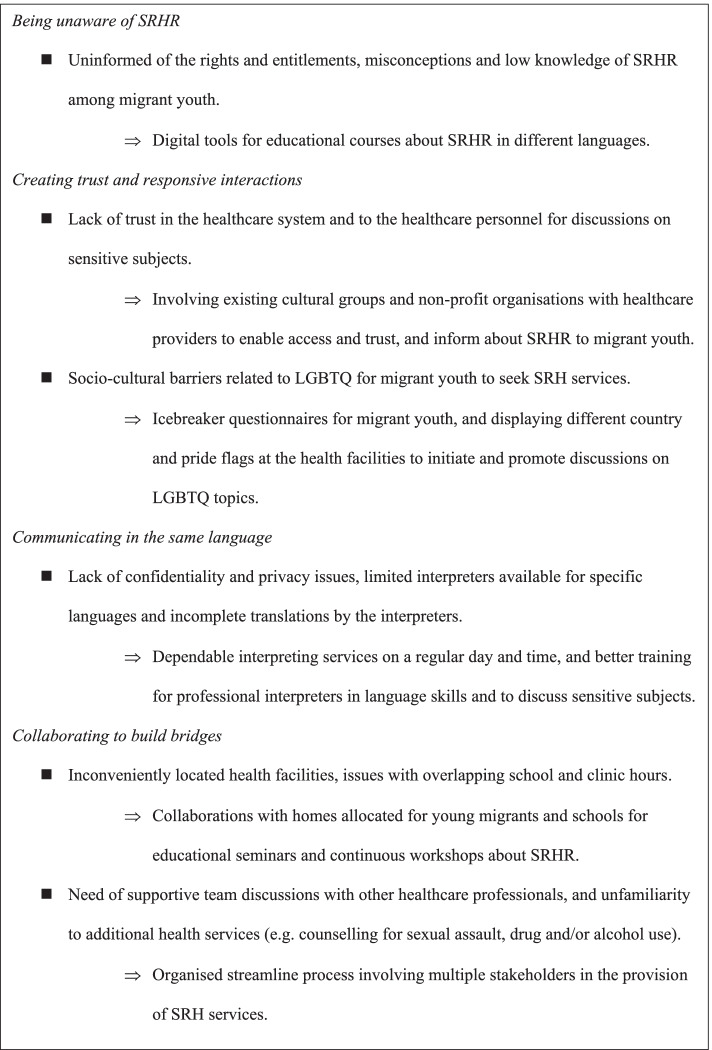
Barriers and strategies for improvement to SRH services for migrant youth

Through the analysis, we identified one theme: *Improving the fragmentation in the SRH services* and four sub-themes: being unaware of SRHR; creating trust and responsive interactions; communicating in the same language; and collaborating to build bridges. We formulated the theme and sub-themes as individual statements that could be linked to demonstrate how patterns of interaction between healthcare providers and migrant youths could lead to an increase or a decrease in the utilisation of SRH services [46]. To make our results more accessible to health research and policy, we provide a summary of the barriers and strategies for improvement to SRH services for migrant youth (Fig. 2).

### Theme: improving the fragmentation in the SRH services

The fragmentation in the SRH services was described by the participants mostly as gaps in the SRHR knowledge by young migrants, the challenges of communication, and the need for structures to build culturally sensitive and trusting services. Participants were unsure what kind of information should be prioritised for migrants, but expressed a need for responsible entities to provide it. *“Outpatient care, inpatient care, youth-friendly clinics... One needs to know what kind of information is especially for people who come here (to Sweden) and what they should know about, like their rights and obligations in terms of sexual and reproductive health – which ‘package’ is more important to talk about, and that there should be a structure for it (providing information). If this information should be given by the community or healthcare providers, then that is great, but this should also happen nationally and systematically*” (Midwife).

### Sub-theme 1: being unaware of SRHR

This sub-theme describes the lack of awareness among migrant youth to SRHR and varied levels of knowledge about anatomy and contraception, and misconceptions surrounding SRHR. In addition to the health information provided by the national websites, a suggestion was mentioned by a healthcare provider to use mobile platforms. For instance, making available mobile applications with courses and information on SRHR in different languages for migrant youth may complement and fill in knowledge gaps on SRH.

Participants did not believe migrant youth were sufficiently aware of the SRHR in Sweden, and this was explained as one reason behind why young migrants do not seek SRH services. *“It is the rights perspective, that not everyone knows what and which type of care you are entitled to in Sweden”* (Midwife). In order to counteract the lack of awareness of SRHR, participants stated that they repeatedly inform migrant youth about their rights in Sweden and about basic knowledge of SRH. *“I inform them (migrants) over and over about a lot of things - I feel that I have to repeat, since it is not certain that they will remember it (their rights)”* (Nurse).

Low knowledge of basic anatomy among some young migrants was a perceived as a barrier by the participants. *“And then when you talk with them (migrants), you really do notice that the level of knowledge is really low, and they are not asking for more information … their questions are also quite interesting because they also reflect low knowledge about anatomy and physiology”* (Midwife). Another participant also reiterated the low awareness about the body. *“They (migrant youth) have very little knowledge about their own body and about how sexuality works, which of course might make them protect themselves less”* (Midwife).

Participants also expressed that a barrier for migrant youths’ SRHR is little knowledge about contraceptives. *“I met a young 25-year old (migrant) man yesterday and asked him have you ever held or used a condom? Maybe once (he answered). Once! So then I gave him (a condom) in his hands (to illustrate how to open and feel it), and I try to give him information here (at the youth-friendly clinic)”* (Midwife). Not knowing about HIV was a cause for some migrant youth to not test for HIV, according to several participants. *“That is what makes them (young migrants) very scared (of testing for HIV) because not knowing about HIV makes them … they do not know that there are medications that can reduce the risk of HIV-transmission”* (Nurse). Participants perceived that migrant youth sometimes had misconceptions about SRHR. *“It is probably that people think that they have not been (HIV) infected so far, so therefore they cannot be infected later. That many people think they are immune to HIV. That is, they have had sex so much and have never become ill … and believe that they will not be in the future. It is a misconception”* (Midwife).

A suggestion shared by one participant was to use modern digital tools to ease discussions and provide further education on SRHR to migrant youth. *“It surprises me, but every one of these (migrant) boys has a mobile phone and uses it all the time. If we have an application for the phone with educational courses and in different languages, it can be something good to increase their knowledge to SRHR”* (Midwife).

### Sub-theme 2: creating trust and responsive interactions

This sub-theme explains the lack of trust to seek SRH services, uneasy discussions with migrant youth, cultural barriers surrounding SRHR, and initiatives made by healthcare providers to effectively provide SRH services in a responsive manner. The barriers entailed the socio-cultural norms and vulnerability that migrant youth may have experienced, in particular disclosing their sexual orientation. The healthcare providers explained their views and suggestions to establish trust and rapport with groups of migrant youth, such as through the involvement of existing cultural groups and non-profit organisations. The healthcare providers also described how they have culturally-adapted care in their practice, such as the use of questionnaires to address sensitive topics, displaying different country and pride flags at the health facilities.

Participants explained that lack of trust in the healthcare system or healthcare providers may hinder migrant youth from seeking SRH services. *“There is a lack of trust. They (migrants) are afraid of the system … that they don’t seek care and may miss important information. It is difficult to gain that trust and be clear about how our healthcare services works”* (Midwife). Participants also explained that it takes time to generate trust between the healthcare providers and migrant youth and that there is a window of opportunity to discuss their past or ongoing issues. *“But it often takes a long time (to establish trust)... It requires trust to say that they have been subjected to some sexual abuse. It is that they trust us first before they share their story”* (Midwife).

Despite being part of the healthcare providers’ responsibilities to ask migrant youth, discussions about compensation for sex were described as an uncomfortable discussion by the participants. *“If I have concerns, then I have the duty to ask if someone receives compensation for sex. It is often difficult to ask, and difficult to get answers to this”* (Counsellor). Another participant described a case of being unsure to question and discuss with migrant youth about transactional sex. *“I meet a young migrant … he is unable to find a home and has tried on different applications (dating sites) to look for a place to sleep, and he said, “I can live in a place for a month, but they just want sex” … I think it might be some kind of transaction there, that he might not want to have sex with these people, but this can be a vulnerable situation to have somewhere to sleep … Quite so, risky”* (Counsellor).

Certain cultural norms were also perceived as potential barriers by the healthcare providers who found that migrant youth were sometimes unable to disclose their sexual orientation or gender identity until they have reached a later point in life. “*I think they (young migrants) are really tormented by them (norms in the family). It is obvious that they are living in the closet (referring to being gay/LGBTQ) and not being able to be oneself … it must be terribly difficult. Many of the migrants have a pragmatic attitude like … It’s just waiting until I grow-up and then I can live my own life after”* (Midwife).

As part of establishing trust and rapport, the participants explained their suggestions to involve groups of young migrants from migrant associations and non-profit organisations in order to inform and motivate migrant youth to seek SRH services. *“People normally organise themselves in one way or another... there are groups or associations that already exist and we could work with these groups. It is something that I think would be good for our clinic, since we cannot expect young migrants to trust and find us”* (Midwife).

Participants reported to have found means for creating a culturally adapted care to reflect young migrants’ culture during conversations on sensitive subjects, for example by having easy questionnaires that serve as an icebreaker for healthcare providers. *“We know that these are difficult and sensitive topics (referring to SRH) to bring up in a conversation – and therefore we have the tools (a questionnaire) to make it easier for us”* (Midwife). Participants have also taken initiatives to promote discussions on LGBTQ topics by tailoring their health facilities more accessible and gender-neutral. *“We have flags (from different countries) and LGBTQ flags in the waiting room too, and with this, we encourage migrants to come, and trust us to talk to us about this (referring to topics of LGBTQ)”* (Midwife).

### Sub-theme 3: communicating in the same language

Communication is summarised in this sub-theme as the participants’ view on their experiences of conversations with migrant youth and issues with interpreters. The barriers to communicating in the same language included the lack of available interpreters, incomplete translations, and discomfort to discuss certain topics in the presence of an interpreter by both healthcare providers and migrant youth. There were strategies for improvement in this sub-theme, such as available and consistent interpreters, and schedule the same interpreter to meet migrant youth.

Participants expressed issues with not being able to have translations for specific languages during consultations with migrant youth, and this was due to the limited available interpreters for certain languages. *“First hand, we always try to get an on-site interpreter, but there are languages such as Georgian that we need to call Malmö (a city in the south of Sweden) to have a telephone interpreter. Unfortunately, with interpreters, some languages are very, very limited in the number who can interpret”* (Nurse). Another participant affirmed the same language barrier between healthcare providers and migrants. “*Then there are some languages that are very difficult since there may only be one or two interpreters at most available”* (Counsellor).

While having an interpreter is necessary to communicate between healthcare providers and migrant youth, participants said that interpreters did not translate everything in some occasions. *“An interpreter was booked, and I did not say anything about knowing Persian. The interpretation was incorrect. I heard what the patient said (in Persian), so I could write what the patient actually said, but this was not translated by the interpreter”* (Nurse). Another participant explained the uncertainty of what was actually translated. *“The barriers, first of all, language …*. *and then having a professionally-trained interpreter, since you don’t know if the person has actually interpreted what is said*” (Midwife).

One barrier to communicating effectively through the assistance of an interpreter is that migrant youth were sometimes worried to disclose personal matters in the presence of the interpreter. *“I have a young person who is quite good at Swedish and waits to ask his most private questions until the interpreter has left. The reason is that they know the person or may go to the same church or do not know if they have some connection with parents... sometimes the most secret things are said when the interpreter has left, which can be difficult to communicate”* (Midwife). Participants explained that some migrant youth prefer not to speak in front of the interpreter about their sexual preference due to close ties to their culture or community. *“We need interpreters … I think in any case that it can be difficult with an interpreter because they (migrants) do not want to come out as an LGBTQ person in front of the interpreter or is afraid that the interpreter will know someone from the same group”* (Counsellor).

The strategies for improvement mentioned by the participants were mainly to prevent these issues with the interpreters. For example, participants suggested the interpreters can be trained or be familiarised in subjects of sexual health, while also able to provide cultural concordance. *“Interpreters should be trained in issues that have to do with sexual health, this is important”* (Counsellor). Participants also emphasised the value of having an interpreter with key qualities given that they act as an important role to migrant youth. *“If we have a good interpreter, and it is the same interpreter being more engaged than just interpreting, then this person becomes a strong link … the interpreter becomes a very important person (for migrants), in fact.”* (Counsellor).

Some healthcare facilities attempted their own strategies to reduce the issues of unavailable interpreters by having the same interpreter and aligning their schedule with migrant youth to then meet the same interpreter. *“We had a consistent interpreter for a while. We tried to find interpreters that were easy and flexible, and we made lists of available interpreters. For example, we had a woman interpreter … we booked her every Tuesday afternoon for translations in Dari and Persian and we could have a consistent interpreter with the young people. We created our own structure, and I think we have become better at knowing how to handle interpreters”* (Midwife).

### Sub-theme 4: collaborating to build bridges

This sub-theme describes the barriers to access SRH services as explained by the healthcare providers and ‘bridges to improve access’ that they have identified including proximity to SRH services, unanticipated costs, and overlapping clinic hours with school hours. The ‘bridges to strengthen services’ were described by the participants as knowing of other SRH services for migrant youths and supportive discussions with other healthcare personnel. The strategies for improvement explained by healthcare providers in this sub-theme included that health facilities collaborate with the homes for care or residence and schools, as well as a streamline of SRH services with other healthcare staff and health facilities.

Proximity to the health facilities such as youth-friendly clinics can be a hurdle if the clinic is too far for migrant youth and if the clinic is known for a specific identified gender or sexuality, such as mostly men or mostly LGBTQ. *“We do think that the distance might be one reason (why migrants don’t come to the clinic) or maybe that we are known for sexual rights or have to be gay or something too specific”* (Midwife). Another barrier described by the participants was related to unanticipated costs to reach the clinic for migrant youth. *“Sometimes they (migrants) do not have money, for example, to get here (to the clinic) –they have to pay for public transportation (i.e. subway and bus) and other things”* (Nurse). Participants explained there are differences across socially-disadvantaged areas. *“I think in socially disadvantaged areas, they (migrants) seek care to a much lower degree than in more affluent areas, and that definitely is the case here at the youth-friendly clinic”* (Midwife). Migrant youth that attend school face the dilemma between skipping school to attend youth-friendly clinics for SRH services. *“The problem is that a lot of them (youths and migrants) are not able to come to us after school and they can only come to see us during school hour, and so they skip school to come to the clinic and (to resolve this) we give them an excuse letter for missing school”* (Midwife).

A recurring barrier expressed by the participants was their unfamiliarity to other specialised services for migrant youth. *“If someone injects drugs or needs to get out of it, where should they go? I can talk about it of course, but for migrants... I do not know (about services)”* (Counsellor). Despite the healthcare staff’s awareness that there are existing services, they were unsure to this procedure for migrant women and youth. *“For example, (migrant) women who have been raped – they are prioritised. However, for us it is to take samples and provide them with contact information for support groups, but I do not know where to refer them”* (Nurse). Participants described that accompanying migrant youth for specialised services can be convenient if this transfer can be done within the health facility. *“It is convenient, we can take someone by the hand and go to there (to see another counsellor). We have an easy access to such help”* (Midwife).

Not having support or team discussions with other health personnel working with SRH services was expressed by the healthcare providers. *“I am the only midwife here. I have no communication with other midwives, and I meet some (midwives) at the network meetings and such, but we do not talk about these things”* (Midwife). One participant mentioned to not have contact with different organisations when asked if there were supportive actions within the health facility. *“No, nothing, I don’t have contact with other organisations that work with sexual health services”* (Nurse). There were differences in the support within the different facilities, and it was seen as important by the participants to have supportive discussions with other healthcare personnel. *“I get work-related support within the clinic from my co-workers who are other midwives, counsellors and gynaecologists and we also have group discussions for more support with a therapist who comes to meet with the team every other week to talk about different cases and how to handle these matters”* (Midwife).

There were several suggestions and attempts by the participants to find potential solutions. For instance, participants explained that collaborating with the homes for care or residence and schools makes a bridge for migrant youth to seek SRH services and receive information. *“We had group activities, they came to the youth-friendly clinic and learned about things we felt they needed to learn … but back then we collaborate with the homes for care or residence, that was the way the youth-friendly clinics did back then”* (Midwife). Another participant explained how they resolved their issues through reaching personnel at the homes for care or residence. *“There were many youth-friendly clinics that started projects and found ways to reach the young people who were in their area, perhaps through a collaboration with schools and housing. We invested more in reaching out to the staff at the homes for care or residence so that they could also help the young people to find us here (at the clinic)”* (Counsellor).

Schools were also suggested by the participants to be a location for lectures and series on SRHR and about SRH services. *“I think the introductory programme (organised by the Swedish Public Employment Services) has not been successful. The lecture series could work as part of education or school”* (Nurse). The participants suggested another avenue to provide SRHR information in larger rooms at school to capture more young people. *“When it comes to young people, I think the school – it is often up to them to provide SRHR information. They have it easier to gather all young people and give lectures.”* (Nurse).

Having collaboration between healthcare providers and young people through language schools and establishing long-standing workshops were mentioned by a participant as a possible solution for positive effects and provide care to migrant youth*. “I think that would be the dream scenario to go out, not only SFI (Swedish language courses) because you can only target some people … it takes time to actually do it (go to the schools) and to keep on going – to build this”* (Midwife). The same participant elaborated on the idea of a continuous workshop. *“One-time interventions, it doesn’t lead to much, and it’s only something easy to report on and check off … I feel like the dream scenario would be to build this relationship, and that takes more time, but it reaches more people, and it involves building trust”* (Midwife). A participant mentioned another proposed solution to develop an organised and streamlined process where several stakeholders and healthcare professionals are involved. *“As in a screening activity (for STIs) like where you streamline (with other clinics for young people, maternal health). I think it has to be done through schools... it may also be necessary to identify workplaces that employ migrants so that employers are also part of this streamline like a checkbox”* (Midwife).

## Discussion

The healthcare providers in our study described that the level of knowledge of SRHR varied among migrant youth, which may have implications for young migrants to know about eligibility for free STI testing and contraceptives in Sweden. In agreement with previous studies, this knowledge deficiency may put young migrants at greater risk of having an unwanted pregnancy and STIs [23, 31]. It also highlights that SRHR education should be part of early resettlement services for refugees and migrant youth [7]. Knowledge and awareness of SRHR is an important component for people’s ability to act in a protective-health behaviour, especially among young people [47]. At the individual level of the socio-ecological model [7, 40], young migrants as a heterogenous group may have different migration experiences and varying levels of knowledge, which could influence accessing SRH services. For healthcare personnel, migrants’ lack of knowledge about their SRH might also play a role in their trust in the healthcare system [31, 35, 48]. The healthcare providers perceived that distrust in the healthcare system or to healthcare providers acts as a prevalent barrier for migrant youth to seek or utilise SRH services. In Sweden, healthcare and the health workforce are accustomed to a relatively high level of trust [49]. However, a lack of institutional trust has been identified as a common barrier to healthcare utilisation by migrants not only in Sweden [35, 50–52] but also in other high-income countries [48, 53]. Trust with healthcare providers is important, especially for young migrants, as it may also generate trust in the healthcare system as a whole [48].

The healthcare providers described cultural norms and confidentiality issues as the migrant youths’ reasons for non-disclosure of their gender identity and sexual orientation. These findings reiterate the wider literature of cultural clash on SRHR [7, 33, 54–56], including that discussions about SRHR are perceived as a particularly sensitive and private topic. For migrants arriving from countries with very different cultural values and beliefs than those of Sweden, such tabooed or private subjects may have immediate and far-reaching implications for the SRHR of young migrants. Cultural beliefs combined with values may also influence young migrants’ ability to have control over their sexual and reproductive needs, and could delay in seeking healthcare for SRH concerns [7, 26]. The healthcare providers in our study perceived that the process of ‘coming out’ for migrant youth as LGBTQ was often suppressed until a later point in life. It is important for healthcare providers to be aware of the cultural and social sensitivities that migrants, especially young migrants, may have concerning SRH services [7]. Interpreters themselves may also be limited by the cultural norms to discuss or translate sensitive subjects [57]. Interpretation services were provided to migrant youth with limited language proficiency; on some occasions, however, we found that it could be challenging for the healthcare providers to rely on the interpreters for communication. For example, there were frustrations expressed by the healthcare providers about incomplete translations and limited interpreters available for less commonly spoken languages. There were also concerns about the interpreters’ judgement and confidentiality, which led the disclosure of LGBTQ and discussions of difficult subjects with migrant youth occurred after the interpreter had left the health facility. Our findings echo with previous studies highlighting that reliance on untrained interpreters for discussing sensitive subjects of SRHR may result in language discordance, including difficulties for clients to gain comprehensive and accurate health-related information [25, 32, 35, 36]. Interpreting services should provide interpreters with a broader understanding of not only the language but also accuracy of the communication related to SRHR, especially as the interpreters act as an important individual to migrant youth [32, 35].

Young migrants may not be aware of free access to SRH services in Sweden, and as reported in other European countries, the sexual and reproductive healthcare entitlements may not be adequately advertised for migrants [58]. Healthcare providers agreed that long-lasting collaboration with other health facilities and services is necessary for improving SRH services. This may lead to building trust, increasing knowledge and awareness of SRHR, and potentially increasing SRH service utilisation by young migrants. In line with a study conducted in northern Sweden, professionals at youth-friendly clinics perceived collaborations between youth-friendly clinics and schools as a strategic platform to promote the healthcare setting among their potential clients [25]. Our study supports this finding, not only for a cooperation across health facilities but also other agencies that cater to migrant youth. Trust is essential in creating and maintaining common goals and building collaborative relationships. However, it might be more challenging for healthcare providers to build trust with migrant youth whose legal status is uncertain, such as temporary residency or a rejected asylum request. It is also likely that migrants’ lack of familiarity with SRHR in a new setting and exposure to different lifestyles and cultural influences could impact migrant youths’ attitudes and behaviours to seek SRH services. In addition, migrants have cultural and language differences and varying needs reflecting their country origins, socio-economic backgrounds, and religious backgrounds. The insights and perspectives provided by healthcare workers in this study can naturally not be used to generalise the experiences of all young migrants.

The healthcare providers in our study shared their suggested solutions and strategies for the improvement of SRH services, which may also be relevant for developing SRHR interventions and programmes tailored to adolescents and migrants. Among the strategies for improvement, the collaboration between the health personnel and the homes for care or residence, as well as the language schools, may allow a linkage for migrant youth to seek SRHR information and of SRH services in Sweden. In addition, these suggestions for improvement were described as a continuous workshop and process that go beyond one-time interventions. Migration stretches across countries and policy interventions. This requires international and regional cooperation, and partnerships with multi-stakeholders such as communities, civil society, and migrants themselves to be well connected to other services and institutions, recognizing both the individual and structural needs [4]. The collective views of the healthcare providers in our study support that healthcare services for migrants are evolving and that they have attempted or suggested new ways to reach young migrants while continuing to improve the fragmentation in the SRH services.

### Strengths and limitations

Our study is of importance as it presents evidence on healthcare providers’ experiences and perspectives on barriers to providing SRH services in the Swedish healthcare. The healthcare providers offered some suggestions for improving SRH services that may be opportune for the inevitable number of people crossing borders, and when migration and mobility resumes post-pandemic. To the best of our knowledge, this study is the first qualitative focusing on the perspectives of multidisciplinary healthcare providers in Sweden on their experiences of providing healthcare for migrant youth, which goes beyond those working with migrant patients and of youth-friendly services.

Approaches to ensure the trustworthiness of our data were consistent with the accepted practice of supporting objectivity, dependability, credibility, and transferability [44]. There could be barriers or suggestions for improvement that were identified and relevant or applicable in a given context if tailored to the situation. Therefore, drawing key messages from the results and considering their potential transferability can help identify ways to improve the SRH services to migrant youth in different settings. Our focus is on SRHR, although not all domains of SRH health were discussed in our study, such as fertility and reproductive organ cancers or aspects related to psychological well-being. This study is representative of the people working within the healthcare sector with the limitations of not having equally recruited male and female participants of different ages and only capturing a small sample. We initially reached out to health facilities that focused on the provision of SRH services, and then we invited healthcare providers from each of the facilities that had responded to our invitation. Since we conducted a purposive sampling, the interviews continued until we reached information saturation and received no new perspectives [43]. The interviews were carried out by VT, SE, and ISH whom all have a background related to SRHR. This was an advantage for understanding the healthcare providers’ work and potentially enhancing rapport. The interviewers’ identity is influential when interviewing healthcare providers [59], and this may have also influenced the interviewers’ ways of asking and probing questions. Even with these considerations in mind, the healthcare providers were open and sincere, and they provided rich descriptions of their experiences, barriers faced, and strategies for improvement. Ten health facilities did not respond to our invitation for healthcare personnel to participate in our study. Two clinics declined to participate as their clientele consisted of mostly Swedish-native youth. These limitations are, however, expected in an exploratory qualitative design.

## Conclusion

Our study provides an understanding of the complexity of engaging migrant youth in SRH services. It describes the views of healthcare providers, including counsellors, nurses and midwives, and their perceived barriers to SRH services in Sweden. Under-utilisation of SRH services was mostly attributed to this lack of trust and awareness of SRHR, which has been found in other studies focusing on marginalised groups and more vulnerable migrants [25, 60, 61]. SRH services need to be viewed as approachable and accessible, especially for young migrants. While the strategies for improvement mentioned in our study gave examples of good practice, we believe these serve as recommendations, which include involving existing cultural groups and organisations to enable trust, use of culturally-adapted and practical communication techniques, consistent and dependable interpreters, and collaborations with homes allocated for young migrants and language schools for direct linkage to SRH service providers. Our findings suggest that there are possible alternatives for healthcare providers to provide SRH services for migrant youth, and some findings serve as a basis for policy discussion concerning the Swedish healthcare system.

## Data Availability

The datasets generated during this study are not publicly available due to the sensitive and personal nature of the information contained. Data may be available from the corresponding author upon justified request and following the ethical approval.

## Abbreviations

HIV: Human immunodeficiency virus
LGBTQ: Lesbian, gay, bisexual, trans, queer or questioning
SDG: Sustainable development goals
SFI: Swedish language courses
SRH: Sexual and reproductive health
SRHR: Sexual and reproductive health and rights
STI: Sexually transmitted infections
